# Ozone injections reduce pain in knee osteoarthritis: a systematic review and meta-analysis

**DOI:** 10.4103/mgr.MEDGASRES-D-25-00088

**Published:** 2026-01-06

**Authors:** Pedro Iván Arias-Vázquez, Rosa Giannina Castillo-Avila, Karen Hernández-Gil, Mauro Nicolás Guzzardo, Duilio Román Guzzardo

**Affiliations:** 1 Department of Rehabilitation, Juárez Autonomous University of Tabasco, Tabasco, Mexico; 2 Pain Training and Research Team (EFID, for its acronym in Spanish), National University of Rosario, Rosario, Argentina

**Keywords:** anti-inflammatory effect, antioxidant effect, cartilage, corticosteroids, intra-articular injection, knee, osteoarthritis, osteoarthrosis, ozone, oxygen-ozone

## Abstract

The only intra-articular injections recommended by international guidelines for the treatment of knee osteoarthritis are injections of corticosteroids and hyaluronic acid; nonetheless, other substances including ozone, dextrose and platelet-rich plasma are also used in clinical. Intra-articular injections of ozone have been reported to have anti-inflammatory mechanisms and clinical benefits similar to intra-articular injections of corticosteroids in patients with knee osteoarthritis; however, this treatment has not been evaluated in a meta-analysis. The objective of this review is to evaluate the effectiveness of intra-articular injections of ozone for reducing pain and improving function in individuals with knee osteoarthritis when compared with intra-articular injections of corticosteroids. An online search was performed using the electronic databases PubMed, EMBASE, Central Cochrane and Web of Science, for controlled clinical trials that compared intra-articular injections of ozone and intra-articular injections of corticosteroid in the treatment of knee osteoarthritis. Seven clinical trials were included in this review, gathering 409 individuals with knee osteoarthritis. In the pooled analysis, ozone injections were found to be more effective in reducing pain in the short and medium terms than corticosteroids injections. Similarly, function improvement in the medium term was observed in favor of ozone injections. Our results suggest that ozone injections represent a good alternative to corticosteroids injections for reducing pain in the short and medium terms in individuals with knee osteoarthritis. Nonetheless, definitive conclusions could not be drawn due to the limited quality of the included studies. Better quality clinical trials are needed to strengthen the evidence and confirm these results.

## Introduction

Osteoarthritis is the most common rheumatic disease, being the knee, the most frequent location affected,^1^ causing pain and function limitations in individuals who present it.^2^ The multimodal treatment of knee osteoarthritis (KOA) includes several therapeutic elements such as pharmacological and non-pharmacological measures, minimally invasive measures such as intra-articular injections or radiofrequency procedures to genicular nerves, and surgical treatments.^3^,^4^,^5^ The substances most commonly used in intra-articular injections for the treatment of KOA are corticosteroids and hyaluronic acid, both recommended in international guidelines.^4^,^5^ The intra-articular corticosteroid injections (ICI) are highly common for treating KOA.^4^,^5^ In the short term, ICI are an effective intervention for reducing pain in individuals who present KOA.^6^

The molecular mechanisms of ICI involve a decrease in the synthesis of prostaglandins and leukotrienes, the migration of leukocytes and the decrease in the levels of cytokines and proinflammatory enzymes. Consequently, it has been considered as an intra-articular therapy with anti-inflammatory objectives.^7^ Nevertheless, the use of ICI is controversial, since repeated exposure to corticosteroids can generate harmful effects on cartilage.^8^

Several review studies propose intra-articular ozone injections (IOI) as an effective therapeutic option for managing pain in musculoskeletal pathologies.^9^,^10^,^11^ The efficacy of IOI has been evaluated in individuals with KOA and the reports indicate a short-term pain reduction.^12^,^13^ The IOI mechanism of action has been related to a decrease in proinflammatory cytokines and the reduction of local oxidative stress^10^,^14^; therefore, it is also classified as a therapeutic intervention with anti-inflammatory results.^7^

Although some systematic review studies and meta-analyses have evaluated the efficacy of IOI in individuals with KOA, these reviews have compared it against various interventions including oral pharmacological treatment, physical therapy programs or intra-articular injections of hyaluronic acid, platelet-rich plasma, dextrose, local anesthetics or placebo.^12^,^13^,^15^,^16^,^17^,^18^ The efficacy of ozone injections compared with corticosteroid injections in reducing musculoskeletal pain has been evaluated, but the pooled analysis included several musculoskeletal conditions.^19^ To date, no review studies that specifically compare IOI with ICI in individuals with KOA have been conducted. Therefore, we considered it necessary to conduct an updated review on the efficacy of IOI in the treatment of KOA, compared with ICI, considering that both could have a similar mechanism of action.

To determine if IOI could represent a therapeutic alternative to ICI, this study evaluated its effectiveness against ICI in reducing pain and improving function in individuals with KOA.

## Methodology

This meta-analysis was conducted following the Preferred Reporting Items for Systematic reviews and Meta-Analyses (PRISMA) 2020 guidelines.^20^ It was registered and authorized by the International Prospective Register of Systematic Reviews (PROSPERO) under protocol number CRD420251003128 on March 4, 2025.

The PICOS strategy used in the study is described as: (P) Patients: individuals with clinical and/or imaging diagnosis of KOA, who referred pain and activity limitations; (I) Intervention: IOI; (C) Control: ICI; (O) Outcomes: efficacy in reducing pain and improving function; (S) Study design: randomized clinical trials.

### Search strategy

A systematic search was conducted in the electronic databases of the National Library of Medicine (Medline/PubMed), Cochrane Central Register of Controlled Trials (CENTRAL), Excerpta Medica Data Base (EMBASE), and Web of Science up to February 2025 to identify publications of interest. Additionally, online sources like Google Scholar and Semantic Scholar were searched to identify peer-reviewed manuscripts published outside of indexed databases. The search terminology included Medical Subject Headings (MeSH) terms, entry terms and related terms: [(ozone or ozone-therapy or ozone-injection) AND (knee osteoarthritis)], with multiple combinations between them. No language restrictions were imposed. The complete search strategy is outlined in **Additional Table 1**.

**Additional Table 1:** The search strategies and databases used in the scoping review

Database	Search terms	Number of manuscripts located
National Library of Medicine (Medline/PubMed)	(("ozone"[mesh] or ozon*[tiab]) AND (“osteoarthritis, knee” [mesh] or “knee osteoarthritis”))	70
	((ozone or “oxygen – ozone” or “ozone – therapy” or “ozone - injection”) AND (“knee osteoarthritis”))	79
Excerpta Medica Data Base (EMBASE)	((exp ozone/ or ozon*.mp.) AND (exp ‘knee osteoarthritis’))	92
Cochrane Central Register of Controlled Trials (CENTRAL)	((ozone or “oxygen – ozone” or “ozone – therapy” or “ozone - injection”) AND (“knee osteoarthritis”))	79
Web of Science	((ozone or “oxygen – ozone” or “ozone – therapy” or “ozone - injection”) AND (“knee osteoarthritis”))	100
Google Academic	((ozone or “oxygen – ozone” or “ozone – therapy” or “ozone - injection”) AND (“knee osteoarthritis”))	238
Total		658

### Types of studies

Inclusion studies: Randomized clinical trials that used IOI for treating individuals with KOA and compared them with ICI.

Excluded studies: Other study designs, including comparative observational studies, case series, one-case reports, preclinical experimental studies, review studies, clinical comments, and editorials. Additionally, clinical trials comparing IOI with interventions other than ICI were excluded.

Eligible studies were required to clearly describe their interventions, evaluation methods, and reported outcomes.

### Participants

Inclusion criteria: (1) Subjects diagnosed with KOA, established by clinical and/or imaging evaluation, referred pain and function limitations; (2) Aged 18 years or older; (3) Both sexes.

Exclusion criteria: (1) Recent history of knee trauma or infection, (2) History of knee surgery, (3) Presence of inflammatory rheumatic diseases or uncontrolled metabolic disorders, (4) Anticoagulant treatment or coagulation disorders, (5) Contraindications to ozone application: glucose-6-phosphate dehydrogenase deficiency, uncontrolled hyperthyroidism, leukemia.

### Intervention characteristics

Ozone group: Studies included individuals treated with one or more sessions of IOI, administered alone or in combination with local anesthetics.

Control group (corticosteroids): Studies included individuals treated with one or more sessions of ICI, administered alone or in combination with local anesthetics.

For both groups, therapeutic procedures were required to be performed either using the anatomical technique or under ultrasound guidance, provided the same technique was consistently applied across all comparative groups within a study. Similarly, co-interventions were permitted only if they were uniform across all groups.

### Evaluation of the risk of bias

Following the Cochrane recommendations for Systematic Reviews, the RoB-1.0 scale was used to assess the risk of bias.^21^ The scale includes seven domains, each categorized as low, high, or unclear risk of bias. The risk of bias of the trials was classified into three categories: a) low (when all domains were rated as low risk); b) unclear (when one or two domains were rated as high or unclear risk); and c) high (when three or more domains were rated as high or unclear risk).

The risk of bias from included studies was independently evaluated by two researchers (PIAV and RGCA). A third reviewer (MNG) arbitrated any discrepancies. The quality of evidence for the evaluated outcomes was determined using the Grading of Recommendations, Assessment, Development and Evaluation system (GRADE) methodology.^22^

### Assessment of study eligibility and data extraction

Titles, abstracts, and full studies were independently screened by two reviewers (PIAV and RGCA) who then determined study eligibility. Discrepancies regarding study inclusion were resolved by consensus with a third reviewer (MNG). Finally, data from the included studies were independently extracted by the three researchers (PAV, RGCA and MNG).

### Outcomes

The efficacy of the interventions was assessed through two primary outcomes: pain reduction and functional improvement.

The pain reduction was measured using validated pain measurement scales such as the visual analogue scale^23^ or numerical rating scale,^23^ as well as the pain subscale score of validated questionnaires.^24^

The improvement in function was measured through validated questionnaires such as the Osteoarthritis Index of Western Ontario and McMaster University,^24^ the Knee injury and Osteoarthritis Outcome Score,^24^ the Oxford Knee Scale^24^ and the Oswestry Disability Index.^25^

Pain reduction and function improvement were evaluated according to the time of follow-up in the short (4–6 weeks), medium (8–12 weeks) and long terms (> to 24 weeks).

Adverse effects were considered secondary outcomes and were described based on data from the included studies.

### Statistical analysis

The Review Manager 5.4 Software (Copenhagen: The Nordic Cochrane Centre, The Cochrane Collaboration, 2014) was used to perform statistical analysis.

The efficacy of the interventions was evaluated by analyzing the reduction of pain and improvement in function according to the follow-up time.

The effect magnitude for pain reduction was calculated comparing the ozone group with the control group, evaluating the change according to what was reported for each follow-up period in the included studies.

The effect magnitude for function improvement was calculated comparing the ozone group with the control group, via evaluating the changes reported for each follow-up period in the included studies. When the scales did not measure improvement in the same direction, the values of the discordant scale were adjusted using the formula (maximum value of the scale - reported value of the scale); with this adjustment, we ensured that all the scales measured improvement in the same direction.

For studies reporting results in formats other than mean and standard deviation, statistical calculators (e.g., RevMan Calculator, The Nordic Cochrane Centre, The Cochrane Collaboration) were employed for conversion. If direct conversion was not feasible, data were extracted from published figures and graphs or obtained via direct request from the study authors.

The effect magnitude was measured using standardized mean difference (SMD) and 95% confidence intervals (CI). A random-effects model was used for combining data, considering the statistical and clinical heterogeneity of the included studies.

Statistical heterogeneity among the studies was assessed using the *I*^2^ and Chi-square tests, along with τ^2^. In this study, statistical heterogeneity was specifically defined by the following parameters: *I*^2^ > 50%, τ^2^ > 0, or a *P*-value < 0.10 in the Chi-square test for heterogeneity. Additionally, a sensitivity analysis was performed to assess the stability of the meta-analysis results. This involved excluding studies with lower statistical weight and eliminating studies with a larger effect size favoring either ozone or corticosteroid injections, or studies deemed to have a high risk of bias.

Publication bias analysis was not performed, as the number of included studies was fewer than 10.

For the evaluation of adverse effects, descriptive measures were used to summarize the data provided in the included studies.

## Results

### Search results

The initial electronic literature search, conducted using MeSH terms and established keywords, identified 658 articles. After duplicate removal, 465 records were excluded, leaving 193 unique articles for screening. Subsequently, the titles and abstracts of these 193 articles were screened, leading to the exclusion of 127 manuscripts. Reasons for exclusion at this stage included studies on ozone therapy in non-musculoskeletal pathology, animal models, ambient ozone, and other topics unrelated to the review’s scope.

**Figure 1:**
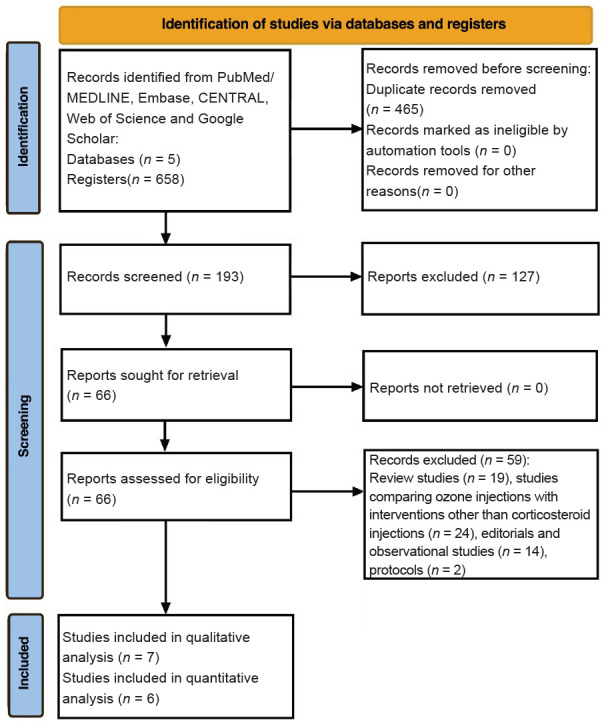
Systematic review flow diagram. A total of 658 citations were identified in all databases; 465 duplicates were excluded. 193 studies were reviewed, of which 186 were excluded. Finally, 7 studies were eligible for inclusion in the systematic review.

Of the remaining 66 articles retrieved for full-text assessment, 59 were further excluded for various reasons: review articles (*n* = 19), studies comparing IOI with interventions other than ICI (*n* = 24), observational studies and editorials (*n* = 14), and study protocols (*n* = 2). Finally, seven clinical trials were included in the qualitative synthesis,^26^,^27^,^28^,^29^,^30^,^31^,^32^ and six of these trials were included in the quantitative analysis.^27^,^28^,^29^,^30^,^31^,^32^ The flowchart of the systematized search is shown in **Figure 1**.

### Study characteristics

**Figure 2:**
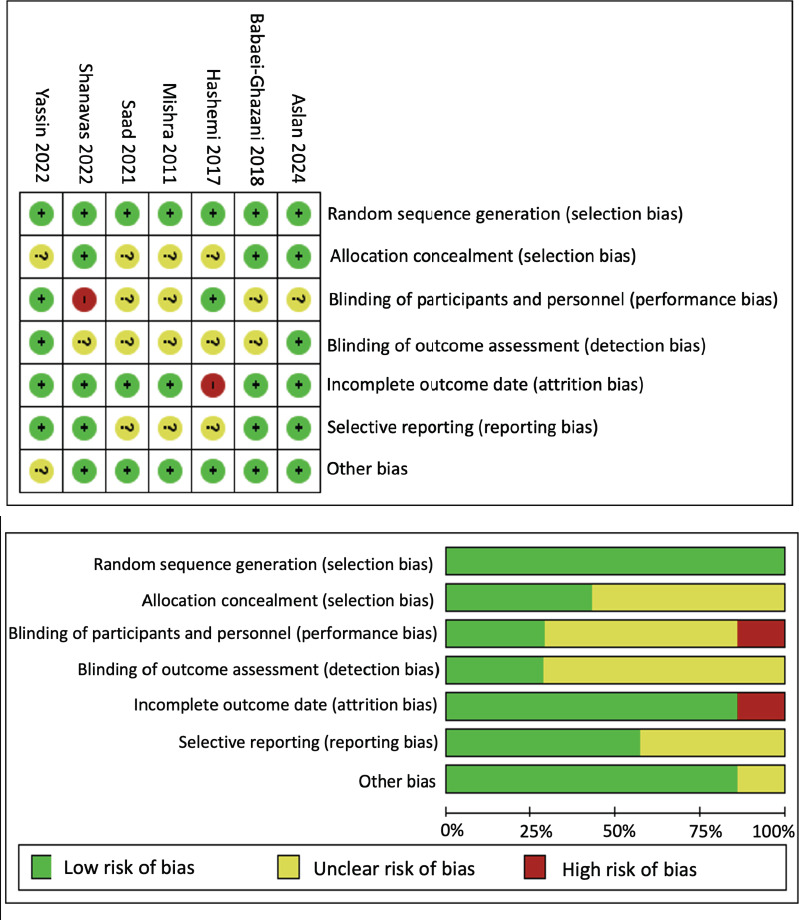
Summary assessment of the risk of bias of the included clinical trials. Four clinical trials had unclear risk of bias and three studies had a high risk of bias, presenting limitations mainly in domains related to selection bias, performance bias and detection bias.

Seven studies^26^,^27^,^28^,^29^,^30^,^31^,^32^ that compared IOI with ICI in individuals with KOA were included in the qualitative analysis. In the risk of bias analysis with RoB-1.0 scale, four clinical trials^28^,^30^,^31^,^32^ had unclear risk of bias, and three studies^26^,^27^,^29^ had a high risk of bias (**Figure 2**).

Six studies conducted short-term follow-ups,^27^,^28^,^29^,^30^,^31^,^32^ six studies performed medium-term follow-ups^26^,^27^,^28^,^30^,^31^,^32^ and three studies performed long-term follow-ups.^27^,^29^,^30^

### Demographic characteristics

A total of 409 individuals diagnosed with KOA met the inclusion criteria and were included in this systematic review. Of these, 205 individuals received IOI and had an average age of 59.2 years, while 204 participants received ICI with an average age of 57.7 years.

### Treatment characteristics

Four studies applied IOI only once^27^,^28^,^29^,^31^; while in three studies, three injections were applied with a weekly frequency.^26^,^30^,^32^ The ozone group used 10 to 35 μg/dL oxygen–ozone mixture,^26^,^27^,^28^,^29^,^30^,^31^,^32^ finding a statistical mode of 30 μg/dL.^26^,^30^ These oxygen-ozone mixtures were administered in volumes ranging from 5 to 10 mL per injection,^26^,^32^ with 10 mL being the mode.^26^,^28^,^30^,^31^,^32^

In the control group, methylprednisolone,^26^,^29^,^31^ triamcinolone^27^,^28^ or betamethasone^30^,^32^ were also used. In the majority of studies, ICI were applied only once.^26^,^27^,^28^,^29^,^31^,^32^ In one study, three ICI were applied with a frequency of every 2 weeks.^30^

In three studies, the use of cointerventions was reported in both groups.^26^,^28^,^29^ In the remaining studies, the use of cointerventions was not reported.

Four studies reported no major adverse reactions in both groups,^27^,^28^,^31^,^32^ only erythema and moderate transient pain that did not require any type of treatment in ozone group.^31^ However, one case of septic arthritis was reported in one patient treated with ozone who had a history of chronic osteomyelitis.^30^ Two studies did not report the presence or absence of adverse reactions.^26^,^29^

**Table 1** presents intervention characteristics and results of the included studies.

**Table 1:** Characteristics, interventions and results of the included studies

Author	Year	Study design	Ozone group	Control group	Outcomes	Adverse reactions/side effects
Mishra et al.^26^	2011	46 patients with KL grade < II knee OA were enrolled in a randomized clinical trial and assigned to two groups.	23 patients (42 ± 4 yr) received three monthly intra-articular injections of 10 mL of ozone (30 μg/mL concentration) and 2 mL of lidocaine.	23 patients (42 ± 4 yr) received an intra-articular injection of 40 mg of methylprednisolone and 2 mL of lidocaine.	Percentage of patients with observed pain reduction (WOMAC pain subscale) and improved function (WOMAC disability subscale) at 3-mon follow-up. In the between-group analysis, no statistically significant differences were observed in pain reduction and functional improvement at 3-mon follow-up.	Not reported.
Hashemi et al.^27^	2017	61 patients diagnosed with KL grade II knee OA were included in a randomized clinical trial and divided into two groups.	30 patients (aged 56.7 ± 16.9 yr, BMI 22.6 ± 4.3 kg/m^2^) received intra-articular injections of 5 mL of ozone (35 μg/mL concentration).	31 patients (aged 42 ± 4 yr, BMI 21.4 ± 3.83 kg/m2) received an intra-articular injection of 50 mg (5 mL) of triamcinolone.	Decrease in pain and improvement in function. Within-group analysis showed a statistically significant reduction in pain and improvement in function at 1, 3, and 6 mon of follow-up in both groups. In the between-group analysis, statistically significant differences were observed in favor of the ozone group for pain reduction and functional improvement at 3 and 6 mon.	No adverse effects were observed in either group.
Babaei-Ghazani et al.^28^	2018	62 patients with KL grade I-III knee OA were enrolled in a randomized clinical trial and assigned to two groups.	31 patients (aged 59.64 ± 10.24 yr, BMI 28.83 ± 2.46 kg/m^2^) received intra-articular injections of 10 mL of ozone (15 μg/mL concentration).	31 patients (aged 56.26 ± 7.88 yr, BMI 29.22 ± 4.53 kg/m^2^) received intra-articular injections of 40 mg of triamcinolone.	Decrease in pain and improvement in function. Within-group analysis showed a statistically significant reduction in pain and improvement in function in both groups at 1 wk, 1 and 3 mon of follow-up.	No adverse effects were observed in either group.
Saad et al.^29^	2021	75 patients diagnosed with KL grade III-IV knee OA, were included in a randomized clinical trial and divided into three treatment groups.	25 patients (aged 69 ± 8 yr, BMI 30 ± 4 kg/m^2^) received intra-articular injections of 5 mL of ozone and radiofrequency ablation of the genicular nerves.	25 patients (aged 63 ± 12 yr, BMI 33 ± 7 kg/m^2^) received an intra-articular injection of 40 mg methylprednisolone and radiofrequency ablation of the genicular nerves. 25 patients (aged 65 ± 14 yr, BMI 33 ± 7 kg/m^2^) received only radiofrequency ablation of the genicular nerves.	Decrease in pain, measured by the VAS, and improvement in function, assessed by the Oxford Knee Score. Within-group analysis showed a statistically significant reduction in pain and improvement in function at 1 wk, 1 and 6 mon of follow-up across all three treatment groups. In the between-group analysis, a statistically significant difference in pain reduction favoring the ozone group was observed at 1 wk of follow-up. No other statistically significant between-group differences were found.	Not reported.
Yassin et al.^30^	2022	40 patients diagnosed with KL grade II-III knee OA were included in a randomized clinical trial and divided into two treatment groups.	20 patients (71 ± 7 yr) received three weekly intra-articular injections of 10 mL of ozone (30 μg/mL concentration).	20 patients (73 ± 4.6 yr) received three intra-articular injections of 2 mL of betamethasone (5 mg/mL) every 2 wk.	Decrease in pain, measured by the VAS, and improvement in function, assessed by the Knee injury and Osteoarthritis Outcome Score. Within-group analysis showed a statistically significant reduction in pain and improvement in function at 6, 12, and 24 wk of follow-up in both groups. In the between-group analysis, statistically significant differences favoring the ozone group were observed for pain reduction at 6, 12, and 24 wk, and for functional improvement at 6 and 12 wk.	A single case of septic knee was presented in the group treated with ozone injections, which had a history of chronic knee osteomyelitis, inactive for 1 yr.
Meethal et al.^31^	2022	54 patients diagnosed with KL grade II-III knee OA were enrolled in a controlled clinical trial and subsequently allocated to two groups.	27 patients (54 ± 5.4 yr) received an intra-articular injection of 10 mL of ozone (20 μg/mL concentration).	27 patients (5 3 ± 5.3 yr) received an intra-articular injection of 80 mg of methylprednisolone.	Improvement in function, as measured by the WOMAC scale. Within-group analysis showed a statistically significant improvement in function at 1, 4, and 12 wk of follow-up in both groups. In the between-group analysis, a statistically significant difference in functional improvement favoring the ozone group was observed at 1 wk of follow-up. No statistically significant differences were found in the remaining between-group measurements.	Pain and mild erythema at the injection site occurred and resolved on its own. No major adverse reactions occurred in either group
Aslan et al.^32^	2024	96 individuals diagnosed with KL grade I-III knee OA were enrolled in this randomized clinical trial and assigned to two groups.	49 patients (aged 62.5 ± 9.5 yr; BMI 29.6 ± 4.6 kg/m^2^) received three weekly intra-articular injections of ozone (10 mL at concentrations of 10, 15, and 20 μg/mL).	47 patients (aged 62.5 ± 8.5 yr, BMI 30.0 ± 5.3 kg/m^2^) received an intra-articular injection of ozone of a mixture of 1 mL of corticosteroid (betamethasone 3 mg/mL) and 1 mL of 2% lidocaine solution.	Pain reduction (VAS) and functional improvement (WOMAC). Within-group analysis showed a statistically significant decrease in pain and improvement in function at 4 and 12 wk of follow-up in both groups. No statistically significant differences were observed in the between-group analysis.	No adverse effects were observed in either group.

BMI: Body mass index; KL: Kellgren-Lawrence; OA: osteoarthritis; RF: radiofrequency; VAS: Visual Analog Scale; WOMAC: Western Ontario and McMaster Universities Osteoarthritis Index.

### Quantitative analysis of pain reduction

Pain reduction efficacy of IOI was evaluated according to follow-up time; this analysis included five short-term,^27^,^28^,^29^,^30^,^32^ four medium-term,^27^,^28^,^30^,^32^ and three long-term follow-up studies.^27^,^29^,^30^

**Figure 3:**
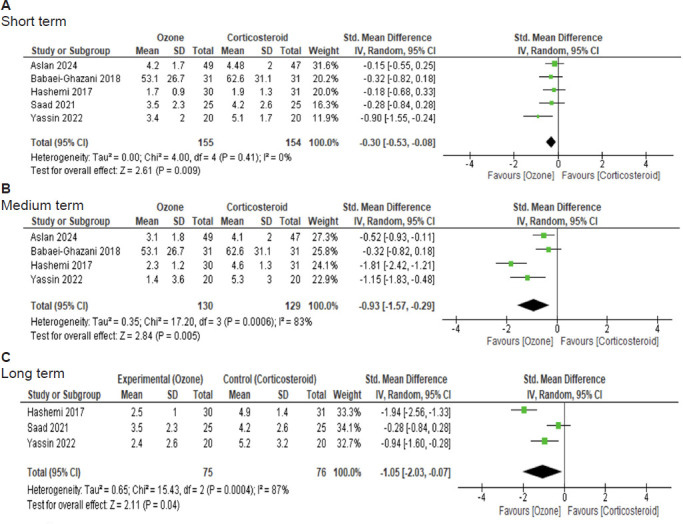
Forest plot of pain reduction of intra-articular ozone and intra-articular corticosteroid injection in patients with knee osteoarthritis. (A) Short term. (B) Medium term. (C) Long term. A statistically significant difference in pain reduction was found in favor of the ozone group in the short, medium and long terms compared with the control group.

In the pooled analysis, a statistically significant difference in pain reduction favoring ozone group was found in the short term (SMD = –0.30, 95% CI: –0.53 to –0.08, *P* (z) = 0.009, *I*^2^ = 0%) (**Figure 3A**). No heterogeneity was observed in the short-term results.

The pooled analysis in the medium-term evaluation also revealed a statistically significant difference in pain reduction favoring the ozone group (SMD = –0.93, 95% CI: –1.57 to –0.29, *P* = 0.005, *I*^2^ = 83%) (**Figure 3B**). Statistical heterogeneity was observed in these medium-term results, prompting a sensitivity analysis. After eliminating the study with a high risk of bias and a larger effect size favoring IOI, heterogeneity was eliminated, and the results remained in favor of the ozone group (SMD = –0.61, 95% CI: –1.02 to –0.19, *P* = 0.004, *I*^2^ = 48%).

In the long-term analysis, a statistically significant difference in pain reduction was found favoring the ozone group (SMD = –1.05, 95% CI: –2.03 to –0.07, *P* (z) = 0.04, *I*^2^ = 87%; **Figure 3C**).

In the long-term results, statistical heterogeneity was observed, however, the small number of studies did not allow for sensitivity analysis.

### Quantitative analysis of improvement in function

The efficacy of IOI for improving function was analyzed according to the follow-up time; it included six studies with a short-term follow-up,^27^,^28^,^29^,^30^,^31^,^32^ five studies with a medium-term follow-up^27^,^28^,^30^,^31^,^32^ and three studies with a long-term follow-up.^27^,^29^,^30^

**Figure 4:**
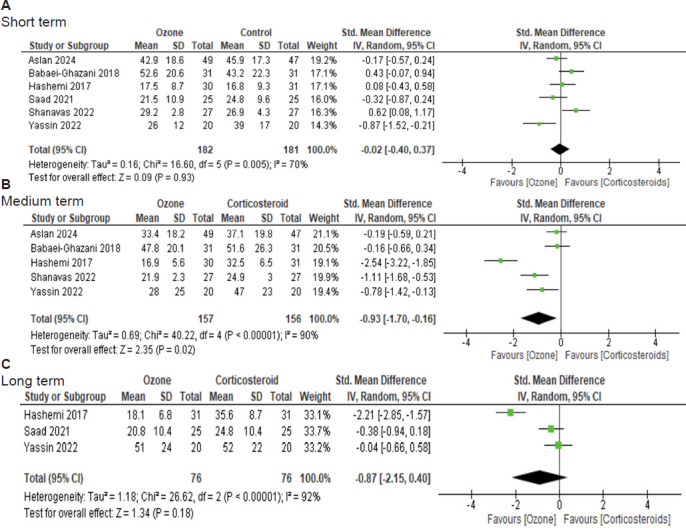
Forest plot of the analysis of functional improvement of intra-articular ozone and intra-articular corticosteroid injection in patients with knee osteoarthritis. (A) Short term. (B) Medium term. (C) Long term. A statistically significant difference in functional improvement was found in favor of the ozone group in the medium term compared to the control group.

The pooled analysis in the short-term evaluation, showed no statistically significant differences in function improvement between ozone and control groups (SMD = –0.02, 95% CI: –0.40 to 0.37, *P* (z) = 0.93, *I*^2^ = 70%; **Figure 4A**).

In the medium-term pooled analysis, a statistically significant difference in function improvement was found in favor of the ozone group (SMD = –0.93, 95% CI: –1.70 to –0.16, *P* (z) = 0.02, *I*^2^ = 90%; **Figure 4B**).

Statistical heterogeneity was observed, and a sensitivity analysis was performed by eliminating the study with a high risk of bias and a larger effect size favoring IOI.^27^ While statistical significance remained in favor of the ozone group in the medium term (SMD = –0.52, 95% CI: –0.97 to –0.08, *P* = 0.02, *I*^2^ = 66%), heterogeneity persisted.

No statistically significant differences in functional improvement were observed between ozone and control groups in the long-term pooled analysis (SMD = –0.87, 95% CI: – 2.15 to 0.40, *P* (z) = 0.18, *I*^2^ = 92%; **Figure 4C**).

## Discussion

The objective of this study was to evaluate the effectiveness of IOI in reducing pain and improving function in individuals with KOA, compared with ICI, to determine if IOI could be proposed as a therapeutic alternative to ICI.

The benefits of IOI in patients with musculoskeletal pain have been described by prior narrative reviews.^9^,^10^ Furthermore, systematic reviews and meta-analyses have reported the short-term efficacy of IOI for pain reduction in patients with KOA.^12^,^13^,^15^,^16^,^17^,^18^ These reviews compared ozone against diverse interventions, including intra-articular hyaluronic acid injections, non-invasive treatments, placebo injections, corticosteroids, or local anesthetics. They consistently reported that the beneficial effects of IOI last 1 to 3 months^12^,^13^,^15^,^16^,^17^,^18^; however, these studies have not specifically compared IOI with ICI.

A recent meta-analysis reported that IOI have lower short-term efficacy but equal medium-term efficacy than ICI for reducing musculoskeletal pain, however, the analysis was performed including various musculoskeletal pathologies in the pooled analysis. Nonetheless, in that meta-analysis, all pathologies were analyzed together, and no analyses by specific pathologies were performed.^19^

In our meta-analysis, seven randomized clinical trials compared the efficacy between IOI and ICI in patients with KOA in terms of pain reduction and function improvement.^26^,^27^,^28^,^29^,^30^,^31^,^32^ Regarding pain reduction, a statistically significant difference was found in favor of the ozone group in the short, medium and long terms compared with the control group (**Figure 3**). Regarding function improvement, a statistically significant difference was found in favor of the ozone group only in the medium term, when compared with the control group (**Figure 4B**).

ICI represent a therapeutic intervention that is accepted in international guidelines for the treatment of KOA.^4^,^5^ Furthermore, several meta-analyses have demonstrated their short-term efficacy in reducing pain and inflammation in patients with KOA.^6^ It has also been proposed, however, that the repeated use of ICI could generate adverse effects on cartilage.^8^,^33^,^34^ Additionally, there is some concern regarding systemic deleterious effects.^35^ The aforementioned could be related to their mechanism of action, which is based on decreasing the proinflammatory effects of arachidonic acid, reducing cytokines levels and proinflammatory enzymes.^36^,^37^ Nonetheless, this mechanism also involves the inhibition of growth factors, generating a blocking or slowing effect on tissue repair.^36^,^37^

Although the mechanisms of action of IOI are less studied, they involve inflammation-modulation, intra-articular oxidative stress regulation and antidegenerative effects. Preclinical studies performed in animal models have shown that oxygen-ozone injections in mice with induced arthritis led to a reduction in pro-inflammatory cytokines, including tumor necrosis factor alpha (TNF-α), interleukin (IL)-1β and IL-6, without generating cartilage toxicity.^38^ In an *in vitro* study, Ata et al.^39^ reported that adding ozone at concentrations of 10 and 30 μg to synovial fluid extracted from individuals with KOA, decreased the synovial concentrations of pro-inflammatory cytokines such as TNF-α, IL-1β and IL-6, when compared with synovial fluid without ozone. Similar results have been reported in human clinical studies. Hashemi et al.^27^ reported decreased serum levels of IL-1β and TNF-α in patients with KOA who were treated with IOI. Likewise, Fernández-Cuadros et al.^40^ published that IOI decreased the serum levels of IL-6 in patients with KOA.

Another effect reported when using IOI, is the re-establishment of cellular redox balance with reduction of local oxidative stress, observed in preclinical studies performed in rats with arthritis model,^41^ as well as in *in vitro* study, reporting that the addition of ozone to the synovial fluid of individuals with KOA improved the antioxidant status of the synovial fluid.^39^ This has also been reported in clinical studies performed in humans with KOA, where the modification of intra-articular oxidative stress was demonstrated.^42^ The observed reduction in inflammation markers and oxidative stress following ozone treatment may be associated with the activation of nuclear factor erythroid 2.^43^,^44^

It has also been reported that ozone has anti-degenerative effects on cartilage. In an in vitro study, Sun et al.^45^ observed that using ozone at concentrations of 30 μg/mL decreased the levels of IL-6, TNF-α, matrix metalloproteinase-3 and matrix metalloproteinase-13; improved the viability and activated autophagy of chondrocytes by activating the peroxisomal proliferator-activated receptors gamma and the mammalian target of rapamycin signaling, in chondrocyte cultures treated with IL-1β. Likewise, ozone decreased the matrix metalloproteinase-1 expression, and bone morphogenetic protein-2 expression^46^ and it slowed the process of degeneration of articular cartilage,^47^ when applied in an animal model with osteoarthritis. This antidegenerative effect was also reported by Fernandez-Cuadros et al.^40^ in humans with KOA, where IOI slightly increased the serum concentration of insulin-like growth factor 1.

Both IOI and ICI exert a therapeutic effect based on anti-inflammatory effects.^27^,^36^,^37^ IOI could have additional benefits such as the modulation of intra-articular oxidative stress^42^ and the anti-degenerative effect on cartilage,^40^ unlike corticosteroids that could even generate deleterious effects on cartilage with repeated exposures.^8^,^33^,^34^

Some studies have used synergistic injections of ozone and corticosteroids and have reported additional benefits compared with the use of only one of them.^47^,^48^,^49^ This synergistic effect may involve the reduction of proinflammatory cytokines in addition to the additional modulating effects of oxidative stress and antidegenerative effects that ozone would provide.

On the other hand, IOI may be more compatible than ICI to achieve a synergistic effect with other intra-articular therapies with antidegenerative or trophic effects, such as hyaluronic acid, platelet-rich plasma or dextrose,^7^ since IOI does not suppress the release of growth factors, as it can happen with ICI.^36^,^37^ Synergistic effects have been reported when combining ozone with hyaluronic acid,^50^ platelet-rich plasma^51^ or dextrose^52^ in the treatment of individuals with KOA.

The results of our meta-analysis indicate that IOI were more effective than ICI for reducing pain in the short and medium terms in individuals with KOA, with a moderate level of evidence on the GRADE scale; similarly, IOI were found to be more effective than ICI for improving movement in the medium term with a low level of evidence on the GRADE scale (**Additional Table 2**).

**Additional Table 2:** GRADE evidence profile: ozone injections *versus* corticosteroid injections in knee osteoarthritis

Outcome	Effect size	Number of studies	Type of studies	Limitations	Inconsistency	Indirectness	Imprecision	Publication bias	Quality of the evidence (GRADE)
Pain reduction	*Short-term*	5	RCT	Serious	No serious	No serious	No serious	Undetected	Moderate ⊕⊕⊕⊝
	SMD = -0.30, 95% CI -0.53 to -0.08, P (z) = 0.009, I^2^ = 0%								
	*Medium-term*	3	RCT	Serious	No serious	No serious	No serious	Undetected	Moderate ⊕⊕⊕⊝
	SMD = -0.61, 95% CI -1.02 to 0.19, P (z) = 0.004, I^2^ = 48%								
	*Long-term*	3	RCT	Serious	Serious	No serious	Serious	Undetected	Very low ⊕⊝⊝⊝
	SMD = -1.05, 95% CI -2.03 to -0.07, P (z) = 0.04, I^2^ = 87%								
Improved functionality	*Short-term*	6	RCT	Serious	Serious	No serious	No serious	Undetected	Low ⊕⊕⊝⊝
	SMD = -0.02, 95% CI -0.40 to 0.37, P (z) = 0.93, I^2^ = 70%								
	*Medium-term*	4	RCT	Serious	Serious	No serious	No serious	Undetected	Low ⊕⊕⊝⊝
	SMD = -0.52, 95% CI -0.97 to -0.08, P (z) = 0.02, I^2^ = 66%								
	*Long-term*	3	RCT	Serious	Serious	No serious	Serious	Undetected	Very low ⊕⊝⊝⊝
	SMD = -0.87, 95% CI -2.15 to 0.40, p (z) = 0.18, I^2^ = 92%								

CI: Confidence interval; GRADE: Grading of Recommendations Assessment, Development, and Evaluation; RCT: randomized clinical trial; SMD: standardized mean difference.

Based on the diverse mechanisms of action and the scientific evidence from this meta-analysis, IOI may represent a viable therapeutic alternative to ICI for reducing pain and improving short- and medium-term function in individuals with KOA.

Due to the presence of certain limitations, our results should be taken with reservations. Only seven studies were included in this systematic review, resulting in 409 individuals analyzed, which represents a low number of included individuals. Two of the studies included in the meta-analysis had a high risk of bias, and four had an unclear risk of bias; this may be the main reason why the IOI failed to achieve a strong level of scientific evidence.^53^ However, the GRADE analysis indicated a moderate quality of evidence for short- and medium-term pain reduction.

Most of the included studies reported no serious adverse reactions in the treated individuals in the ozone group^27^,^28^,^31^,^32^; only transient moderate pain (that resolved rapidly) was observed, which is common with IOI treatments, and it has been reported in other systematic reviews.^15^,^18^ However, in the study by Yassin et al.,^30^ a case of septic arthritis was reported in one patient treated with ozone who had a history of chronic osteomyelitis, which had been inactive for 1 year prior to the injection; the authors determined that the presence of the infection may have been due to a reactivation of the chronic osteomyelitis. The presence of bone or joint infection represents a contraindication for performing an intra-articular injection procedure,^54^ therefore, the complication presented may be more related to the inadequate choice of the patient than to the effects of the substance used.

Another limitation of our study is that the treatment regimens of IOI in individuals with KOA are poorly standardized, with considerable clinical heterogeneity due to substantial differences in frequency of applications and the concentrations of ozone used, which could be the origin of the statistical heterogeneity found.

More clinical trials with good methodological quality, low risk of bias and the standardization in treatment regimens are needed, directly comparing IOI versus ICI in individuals with KOA, to corroborate these results and to establish definitive recommendations. Consequently, the present study offers an exploratory foundation that can guide the expansion of scientific evidence on this subject.

## Additional files:

***Additional Table 1:*** *The search strategies and databases used in the scoping review.*

***Additional Table 2:*** *GRADE evidence profile: ozone injections versus corticosteroid injections in knee osteoarthritis.*

## Data Availability

*All data generated or analyzed during this study are included in the manuscript and its additional files.*
